# Another Cancer Cure

**Published:** 1889-05-18

**Authors:** 


### Within the Wards.

### ANOTHER CANCER CURE.

At the Chelsea Hospital for Women several recent attempts have been made to check the growth of cancer by means of a powerful interrupted voltaic current. The rationale of the treatment consists in this: Cancer-cells are believed to be originally similar to, and developed from, the normal cells of the organ or tissue affected. They are quickly proliferated and of rapid growth, and hence are of low vitality and resisting power. Moreover, they are, so to speak, outside the control of the nervous system, and have not the advantage to be derived from the influence of the trophic nerves. If then they, being of lower vitality than the tissue from which they spring, can by any means be checked in the proliferative and developing process without injury to the normal tissue, a practical cure will be effected. Four persons were operated upon during last, and in January of the present, year. So far as appears at present, there is hope that the treatment may be of service. In some of the cases there seems to have been an arrest of growth in the cancer, with an undoubted diminution of pain, and a decided improvement of the general health. It is much too early to form definite conclusions, and the cases as yet are far too few. But the theory of cure is not unscientific, and it is to be hoped that a good many more patients may be willing to submit to it, and that surgeons who have opportunities will give it a thorough and intelligent trial. Progress in the treatment of cancer has not been rapid. It is desirable that every new method which is founded in any degree on a rational physiology shall be promptly and thoroughly tried, in order that, if successful, it may establish itself in the general practice, but if useless, it may be relegated to that unhappy limbo which has received so many failures in the past.

